# Supplementary material for: Stability and Requirement for Thiamin in a Cell Culture Feed Used to Produce New Biological Entities
Source: Cells. 2023 Jan 16;12(2):334. doi: 10.3390/cells12020334 (PMC9857259; doi:10.3390/cells12020334)
Supplement: Supplementary file 1 [file cells-12-00334-s001.zip › cells-2124686-supplementary.pdf]

Supplementary materials

Figure S1: Example of Tier 1 level identification of thiamin

Experimental data obtained in the feed (green) compared to the data obtained for the standard (blue) for feature m/z = 265.1117, RT = 2.56 min, tier 1, identified as compound **1**.

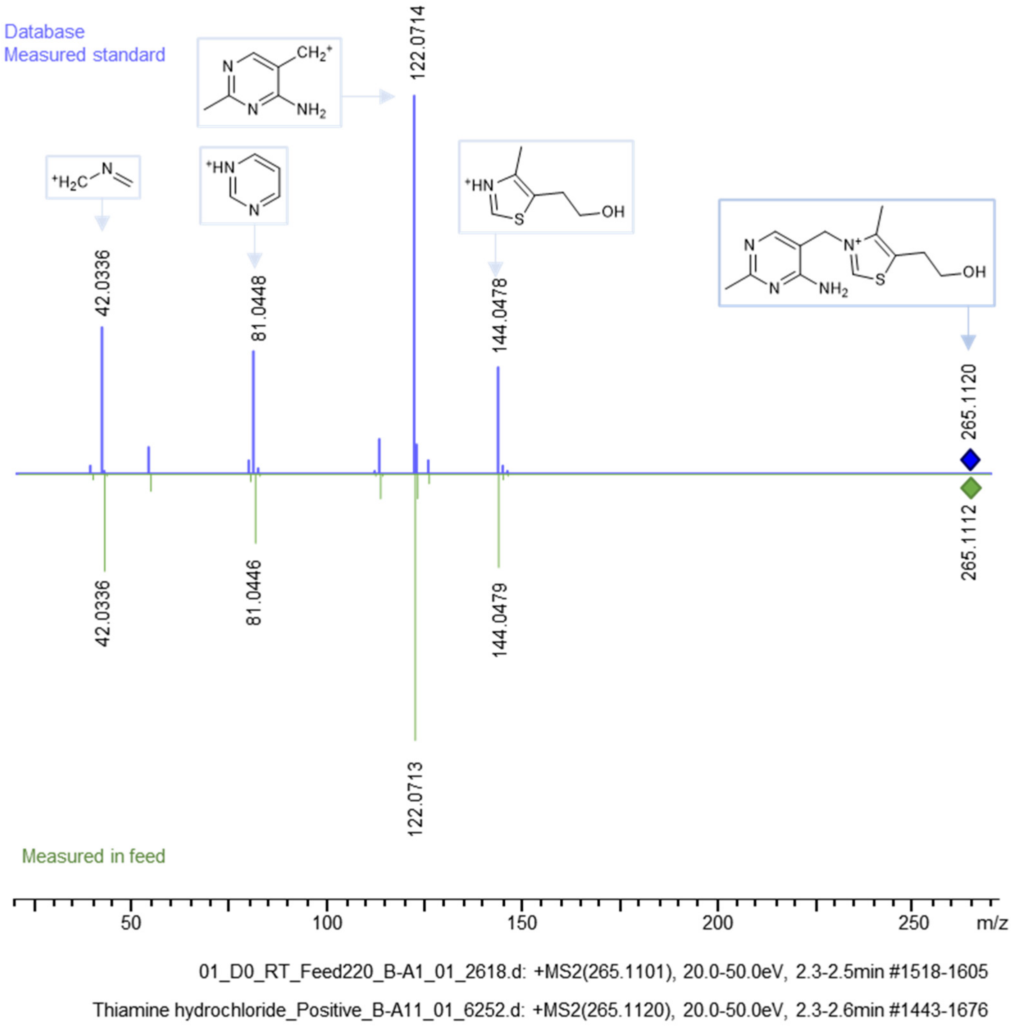

Figure S2: Identification of interaction partner

Heatmap of thiamin degradation products in each component specific deficient feed (-) or feed with addition of specific components (+). PTyr abbreviates phosphotyrosine. Data of vitamin and other component specific deficient feeds are not shown since they were not identified as interaction partner. Light blue color indicates that the feature was not detected at all or only background signal was detected. Change from light to dark blue indicates an increase in feature abundance.

Feature	ID	Tier level	Neutral sum formula	Mass error (ppm)	Maximum Abundance
5.14_267.9879m/z	4	3	C6H9N3O3S3	-1,88	66252
3.47_236.0158m/z	5	3	C6H9N3O3S2	-2,27	27191
4.40_307.0359m/z	6	3	C9H14N4O2S3	0,58	662749
8.06_329.1099m/z	7a	1	C13H20N4O2S2	-1,90	18946841
8.53_329.1100m/z	7b	1	C13H20N4O2S2	-1,80	1355876
8.70_329.1097m/z	7c	1	C13H20N4O2S2	-2,68	3001381
8.85_361.0820m/z	8a	3	C13H20N4O2S3	-1,70	10382043
9.17_361.0823m/z	8b	3	C13H20N4O2S3	-0,87	1614789
9.08_393.0552m/z	9	3	C13H20N4O2S4	1,14	2266972
5.17_363.0789m/z	10	1	C12H18N4O5S2	-2,10	153706
5.88_363.0791m/z	-	4	C12H18N4O5S2	-1,55	86188
6.44_363.0791m/z	-	4	C12H18N4O5S2	-1,55	53483
5.89_395.0514m/z	11	3	C12H18N4O5S3	-0,84	258553
6.63_395.0515m/z	-	4	C24H34N8O4S2	-0,60	27538
7.54_427.0231m/z	12	3	C12H18N4O5S4	-1,65	75364
8.32_343.1266m/z	13	3	C14H22N4O2S2	1,08	1145522
8.54_343.1265m/z	-	4	C14H22N4O2S2	2,58	605301
6.58_444.1733m/z	-	4	C18H29N5O4S2	0,46	849543
6.39_444.1733m/z	-	4	C18H29N5O4S2	0,46	1211545
5.05_402.1266m/z	14a	3	C15H23N5O4S2	-0,96	46587289
5.65_402.1269m/z	14b	3	C15H23N5O4S2	-0,01	4761316
5.48_434.0986m/z	15a	3	C15H23N5O4S3	-0,88	34268790
6.31_434.0991m/z	15c	3	C15H23N5O4S3	-0,06	1268154
6.29_466.0711m/z	16a	3	C15H23N5O4S4	0,09	7952806
7.12_466.0713m/z	16b	3	C15H23N5O4S4	0,44	1082376
7.56_466.0707m/z	16c	3	C15H23N5O4S4	-0,83	172530
7.93_498.0433m/z	17a	3	C15H23N5O4S5	0,38	1784994
8.28_498.0427m/z	17b	3	C15H23N5O4S5	-0,92	142218
5.90_430.1216m/z	18	3	C16H23N5O5S2	-0,71	1748446
5.32_504.1584m/z	19a	3	C19H29N5O7S2	-0,48	59104
5.97_504.1583m/z	19b	3	C19H29N5O7S2	-0,68	941221
7.33_534.1157m/z	20	3	C19H27N5O7S3	1,27	1348016
6.28_502.1439m/z	21	3	C19H27N5O7S2	1,82	2424107
6.19_563.2216m/z	22a	1	C24H34N8O4S2	-0,79	10265027
6.69_563.2228m/z	22b	1	C24H34N8O4S2	-1,14	4482886
6.92_595.1940m/z	23	3	C24H34N8O4S3	-0,53	2678341
7.85_246.0799m/z	24	3	C10H15NO4S	-0,43	25386
4.84_269.1245m/z	25	3	C11H16N4O4	-1,54	14423878
2.03_267.1088m/z	26	3	C11H14N4O4	-1,80	17673868
4.65_249.0984m/z	27	3	C11H12N4O3	-1,49	55271089
4.43_367.1435m/z	28	3	C16H22N4O4S	-1,29	438061
7.34_383.1387m/z	29	3	C16H22N4O5S	-0,60	1848733
2.97_239.1501m/z	30	1	C11H18N4O2	-2,68	1164123
1.61_227.1134m/z	31	1	C9H14N4O3	-4,20	1297728
1.69_254.1244m/z	32	1	C10H15N5O3	-3,65	441160
4.70_251.1137m/z	33	1	C11H14N4O3	-2,78	852774
5.24_253.1662m/z	34	1	C12H20N4O2	-1,08	805080
1.79_241.1296m/z	35	1	C10H16N4O3	-2,00	568898
6.56_287.1504m/z	36	1	C15H18N4O2	-1,42	1245341
2.14_237.1345m/z	37	1	C11H16N4O2	-2,47	666962
2.98_197.1031m/z	38	3	C8H12N4O2	-3,63	238258
5.01_205.1087m/z	39	3	C10H12N4O	-1,22	376523
5.00_253.1660m/z	40	3	C12H20N4O2	-1,63	977215
6.23_263.0961m/z	41	1	C12H14N4OS	-2,12	460174
4.86_266.0963m/z	42	1	C12H15N3O2S	-0,07	16987
1.41_139.0978m/z	43	1	C6H10N4	-3,86	907600
2.23_140.0822m/z	44	1	C6H9N3O	-1,21	300823
7.55_144.0477m/z	45	1	C6H9NOS	-4,10	22063558
6.75_297.0838m/z	46	1	C12H16N4OS2	-2,02	2242051
6.45_281.1064m/z	47	1	C12H16N4O2S	-2,68	856331
2.73_167.0927m/z	48	1	C7H10N4O	-3,63	2478206..
4.52_181.1083m/z	49	1	C8H12N4O	-3,48	1256129
5.98_295.0860m/z	50	3	C12H14N4O3S	-1,66	2963689
2.36_275.0628m/z	51	3	C9H14N4O2S2	-2,84	1831490
1.79_209.1032m/z	52	3	C9H12N4O2	-3,03	2108324
5.08_267.1452m/z	53	3	C12H18N4O3	-1,83	8409538
4.74_239.1136m/z	-	4	C10H14N4O3	-3,08	1832684
5.29_323.0812m/z	-	4	C13H14N4O4S	-0,49	1392503
7.23_438.1587m/z	-	4	C22H23N5O3S	-2,91	167356
8.96_437.1756m/z	-	5	-	-	156908
5.24_282.0531m/z	-	5	-	-	571818
5.47_256.1292m/z	-	5	-	-	358929
7.95_265.1087m/z	-	5	-	-	1104066
8.88_357.1430m/z	-	5	-	-	663166

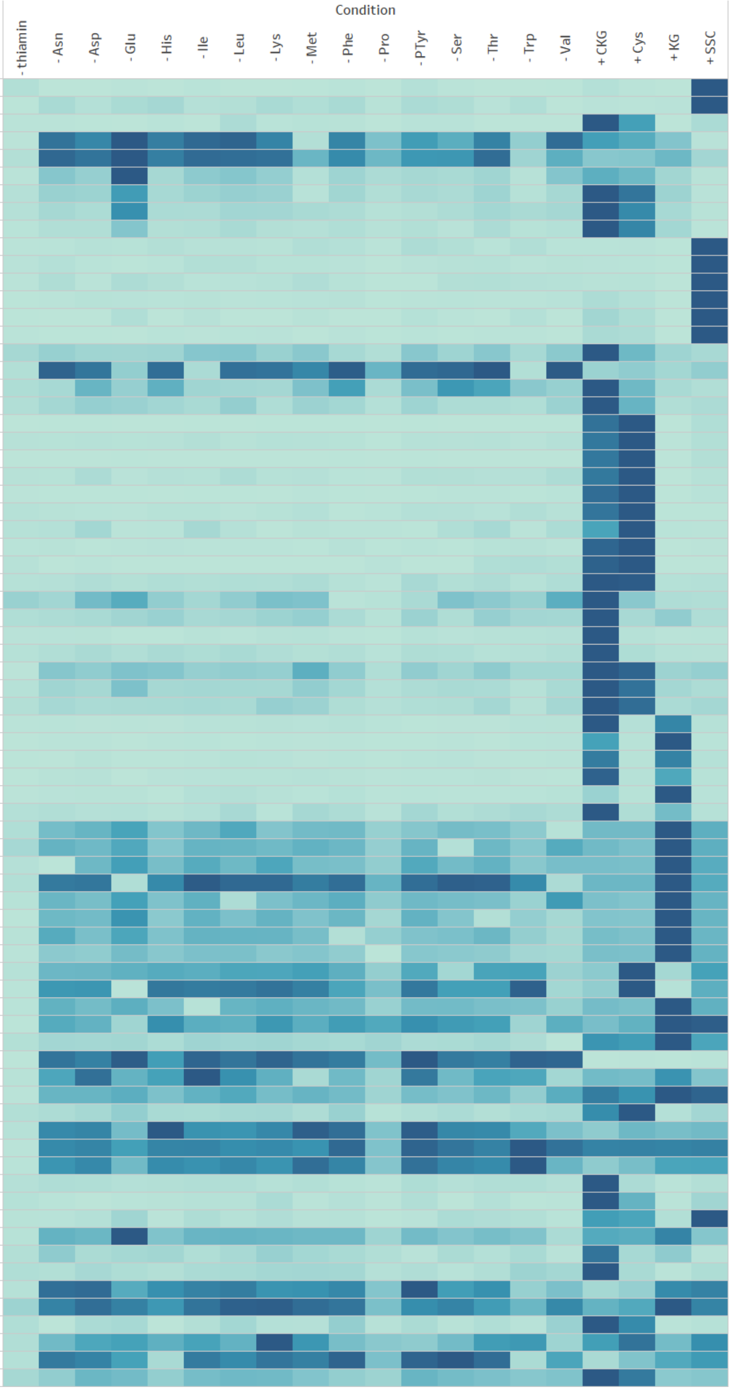

Figure S3: Cell performance of clone 3 and 4 and day 12 cQAs of mAb 3 and 4

Cell performance of clone 3 and 4 during the FB and day 12 cQA analysis of mAb3 and mAb4. A) Viable cell density, B) viability, C) titer, D) relative quantification of intact recombinant protein and total low as well as high molecular weight forms (LMW, HMW), E) relative quantification of charge variants, and F) relative quantification of total fucosylation (Fuc), total high-mannose (Man), total galactosylation (Gal), total sialylation (Sial), and total N-acetylglucosamine (GlcNac) species. $n_{cell\ performance}$=4 and n_{cQAs}=3, error bars represent standard error of mean.

Charge variants of the fusion protein were not characterized because there is no analysis method available in the laboratory yet.

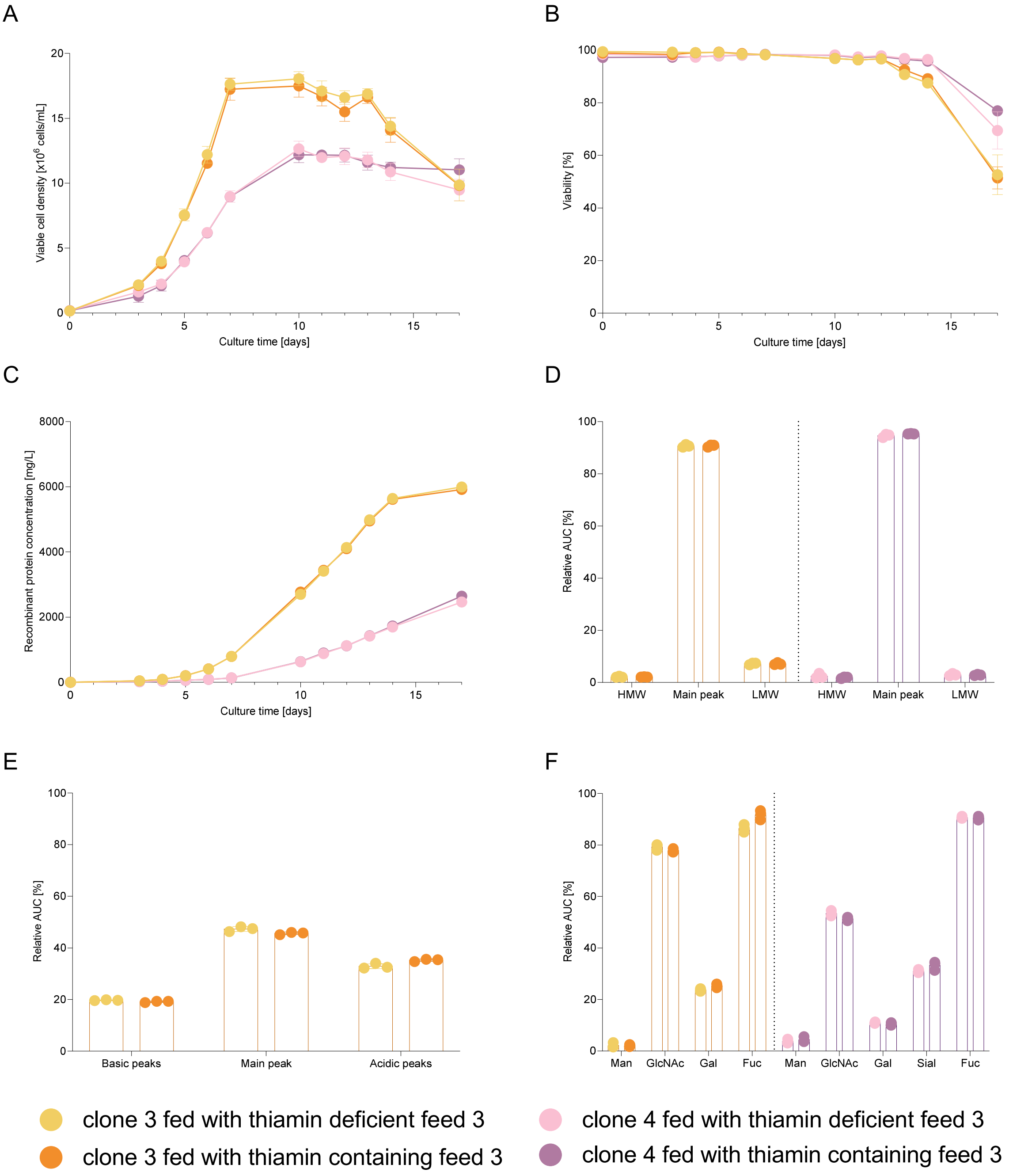

Figure S4: Thiamin phosphate species of clone 3 and 4

Thiamin species detected extra- and intracellularly during the FB. Lighter color indicates the conditions without thiamin in feed 3. Darker color indicates the conditions containing thiamin in feed 3. N=3. Timepoint and lowest thiamin abundance highlighted for conditions fed with a thiamin deficient feed.

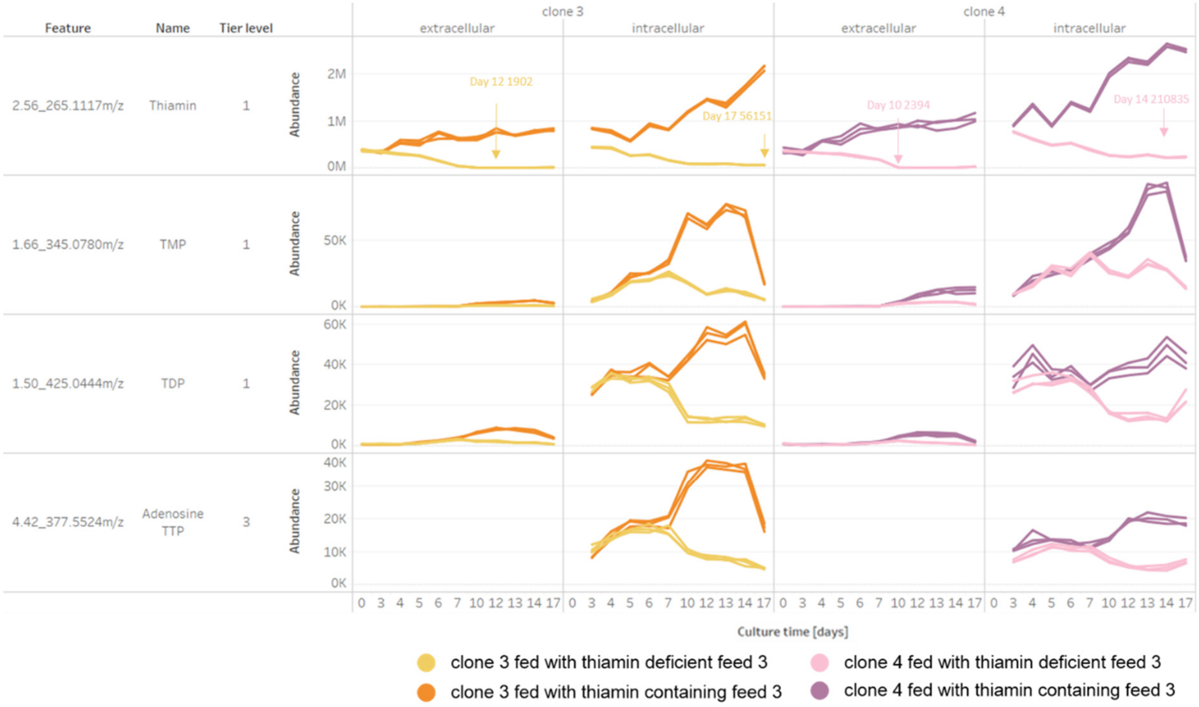

Figure S5: Intracellular and not yet presented features affected by thiamin removal

Structures of features that were not yet presented and found with intracellular metabolomics. Component specific information are presented as [**Identifier**; interaction partner if found or not available (na); tier level]. For **65** and **67** Leu was presented as interaction partner and for **66** and **68** Val was presented as interaction partner. Red highlights structural differences compared to thiamin.

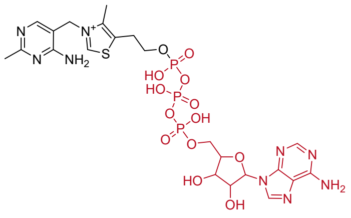

64; ATP; 3

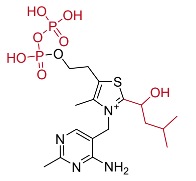

65; Leu; 3

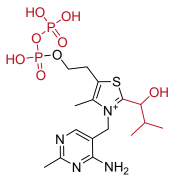

66; Val; 3

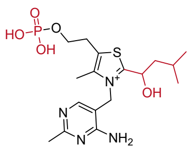

67; Leu; 3

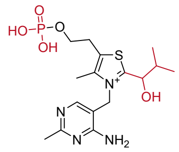

68; Val; 3

Section S1: LC-MS structure/tier assignments for features

Various levels of confidence were assigned to the annotations based on the initial recommendation of Sumner et al. [14] and some further published guidance document [15]. Briefly, tier 1 identification level was given to features where RT, precursor mass, and fragmentation pattern matched a commercially available standard that was measured on the same LC-MS equipment and with the same method. Tier 2 annotations were obtained for features identified by comparing the MS/MS fragmentation pattern with a spectral library (HMDB, METLIN or NIST). Features were annotated as a tier 3 when a tentative structure matched the precursor mass and the MS/MS fragmentation data could be annotated manually. Tier 4 correspond to features for which the precursor mass and the isotopic pattern were matched with a sum formula. Finally, tier 5 were only identified by a unique RT and precursor mass but no tentative structure or sum formula can be reported.

Table S1: Compounds used as standards for tier 1 feature identification.

Description	CAS	ID	# Catalogue	Supplier
Thiamine chloride hydrochloride	67-03-8	**1**	500923	Merck
Thiamin methyl disulfide	2281-20-1	**7**	FCG4202889027	Santai Labs
Thiamine-S-sulfonate	3889-45-0	**10**	CP-19035281	Chempartner
Thiamin disulfide	67-16-3	**22**	T-8410	TLC Standards
2-{[(4-amino-2-methylpyrimidin-5-yl)methyl]amino}-3-methylbutanoic acid	No CAS	**30**	CSCS06256078232	Chemspace
2-{[(4-amino-2-methylpyrimidin-5-yl)methyl]amino}-3-hydroxypropanoic acid	No CAS	**31**	CSC06256078239	Chemspace
2-{[(4-amino-2-methylpyrimidin-5-yl)methyl]amino}-3-carbamoylpropanoic acid	No CAS	**32**	CSCS06256078247	Chemspace
1-[(4-amino-2-methylpyrimidin-5-yl)methyl]-5-oxopyrrolidine-2-carboxylic acid	No CAS	**33**	CSCS06256078234	Chemspace
2-{[(4-amino-2-methylpyrimidin-5-yl)methyl]amino}-3-methylpentanoic acid	No CAS	**34**	CSCS06256078241	Chemspace
2-{[(4-amino-2-methylpyrimidin-5-yl)methyl]amino}-3-hydroxybutanoic acid	No CAS	**35**	CSCS02401040605	Chemspace
2-{[(4-amino-2-methylpyrimidin-5-yl)methyl]amino}-3-phenylpropanoic acid	No CAS	**36**	CSCS06256078209	Chemspace
1-[(4-amino-2-methylpyrimidin-5-yl)methyl]pyrrolidine-3-carboxylic acid	No CAS	**37**	CSMS05241890416	Chemspace
Thiochrome	92-35-3	**41**	T7891	Sigma
Oxythiamin	136-16-3	**42**	O4000	Sigma
4-Amino-5-aminomethyl-2-methylpyrimidine	95-02-3	**43**	QA-1255	Combi-Blocks
Toxopyrimidine	73-67-6	**44**	T686980	Toronto Research Chemicals
Sulfurol	137-00-8	**45**	W320404	Sigma
Thioxothiamine	299-35-4	**46**	PHR1759	Sigma
Oxothiamin	755708-63-5	**47**	375.365	Chemcube
4-Amino-5-(formamidomethyl)-2-methylpyrimidine	1886-34-6	**48**	A609675	Toronto Research Chemicals
N-[(4-Amino-2-methylpyrimidin-5-yl)methyl]acetamide	23676-63-3	**49**	CSC000092869	Chemspace
Thiamin monophosphate chloride dihydrate	273724-21-3	**54**	T8637	Sigma
Thiamine pyrophosphate	154-87-0	**55**	C8754	Sigma
Inosine	58-63-9	**62**	I4125	Sigma

Table S2: Extracellular features affected by thiamin removal

Extracellular metabolomics applied to supernatant during a FB experiment. Area under the curve of interesting feature abundance over time (days 0-17) of thiamin free feed conditions (-) and the respective control condition (+) for all tested clones.

Feature	ID	Tier level	Ion species	Neutral sum formula	Mass error (ppm)	Maximum Abundance	clone 1		clone 2		clone 3		clone 4	
							-	+	-	+	-	+	-	+
2.56_265.1117m/z	1	1	M+	C12H16N4OS	-0,40	1211263	6012257	35381216	6837945	32344724	6142033	31953773	7405114	38734594
6.23_263.0961m/z	41	1	M+H	C12H14N4OS	-2,12	119148	32615	112405	75119	1545730	49446	159461	93085	2051531
3.77_309.1380m/z	57	3	M+	C14H20N4O2S	-1,69	113029	283756	2228717	95496	953453	93475	1559486	90892	900172
5.36_278.6131m/z	-	5	M+2H	-	-	41763	18770	26532	91960	335720	68687	171141	60354	164833
6.97_351.1851m/z	61a	3	M+	C17H26N4O2S	-1,08	30236	203811	667426	121081	340519	102628	352642	137801	367048
4.43_367.1435m/z	28	3	M+H	C16H22N4O4S	-1,29	29372	95727	322285	50215	345648	71305	493850	80365	316088
5.49_307.1224m/z	56	3	M+	C14H18N4O2S	-1,51	19501	95003	322195	65742	218237	74855	289814	67311	183092
5.05_402.1266m/z	14a	3	M+H	C15H23N5O4S2	-0,96	19307	42187	76876	55223	208317	47105	80861	63525	344523
1.66_345.0780m/z	54	1	M+	C12H17N4O4PS	-1,86	13369	23727	48888	79044	270562	26577	86733	76853	233063
5.84_337.1694m/z	60a	3	M+	C16H24N4O2S	-1,19	11423	41375	211167	19810	114512	19387	107273	34075	117993
2.03_267.1088m/z	26	3	M+H	C11H14N4O4	-1,80	11275	72672	138206	99259	224667	95552	250170	82048	198950
5.08_267.1452m/z	53	3	M+H	C12H18N4O3	-1,83	10380	43087	131939	57318	225557	50086	149490	59185	250881

Table S3: Intracellular features affected by thiamin removal

Intracellular metabolomics applied to CHO cell lysates during a FB experiment. Area under the curve of interesting feature abundance over time (days 0-17) of thiamin free feed conditions (-) and the respective control condition (+) for all tested clones.

Feature	ID	Tier level	Ion species	Neutral sum formula	Mass error (ppm)	Maximum Abundance	clone 1		clone 2		clone 3		clone 4	
							-	+	-	+	-	+	-	+
2.56_265.1117m/z	1	1	M+	C12H16N4OS	-0,40	5976252	4338036	31023882	2827786	17828654	2135250	17407778	4827873	26049518
5.39_269.0886m/z	62	1	M+H	C10H12N4O5	0,07	1257920	2407631	2685790	4376782	8307095	2477696	2812657	4716950	8302720
3.77_309.1380m/z	57	3	M+	C14H20N4O2S	-1,69	1042389	375172	4389220	60354	905011	67028	1391287	103537	1539703
2.73_167.0927m/z	48	1	M+H	C7H10N4O	-3,63	659848	1108354	2363571	1369413	2849585	1218757	2636053	1414539	2713394
6.61_402.2119m/z	-	5	M+2H	-	-	430873	1925591	2689069	1300636	2353346	665951	1339066	1798006	2502650
6.27_635.3267m/z	-	5	M+H	-	-	290931	814089	1487748	600025	1616359	232368	687749	901480	1917782
3.66_268.1405m/z	-	5	M+H	-	-	275161	667490	723704	353286	787630	158438	259864	243368	569009
4.43_367.1435m/z	28	3	M+H	C16H22N4O4S	-1,29	206316	201423	1123948	70427	1165169	94744	1412937	163362	1153424
1.97_389.1044m/z	58	3	M+	C14H21N4O5PS	-0,99	183437	710816	1713870	320740	876574	217376	674013	771878	1696562
8.39_534.2547m/z	-	5	M+2H	-	-	169204	677371	991472	517935	1017696	188386	421251	656823	1004866
5.49_307.1224m/z	56	3	M+	C14H18N4O2S	-1,51	158610	124312	769385	22805	265878	25662	385716	47251	353672
5.82_381.7225m/z	-	5	M+2H	-	-	131602	258754	531704	175589	548156	66806	191371	391523	822776
1.50_425.0444m/z	55	1	M+	C12H18N4O7P2S	-0,03	125961	311951	831740	337632	779089	277522	599353	302769	536825
1.71_469.0711m/z	59	3	M+	C14H22N4O8P2S	0,78	120990	477143	973390	319751	503974	270133	409232	393645	566435
8.32_421.2558m/z	-	5	M+H	-	-	120164	531	1273	302	870	82	398	180572	485180
7.23_351.1853m/z	61b	3	M+	C17H26N4O2S	-1,30	109623	102383	350741	5230	26240	9971	42791	18075	48571
1.41_139.0978m/z	43	1	M+H	C6H10N4	-1,21	108612	120295	425691	133409	389163	212454	774723	139632	488359
5.10_348.0712m/z	63	3	M+H	C10H14N5O7P	0,89	106954	273723	335883	299178	705004	192445	370376	356692	549963
5.98_337.1694m/z	60b	3	M+	C16H24N4O2S	-1,19	100221	89507	366697	3232	26936	3110	35533	21081	64118
1.66_345.0780m/z	54	1	M+	C12H17N4O4PS	-1,86	90466	341017	739660	238969	618932	200546	633231	364864	674185
9.13_439.7171m/z	-	5	M+2H	-	-	90039	140997	296246	30382	186932	11494	61238	225641	691527
8.16_253.1657m/z	-	5	M+H	-	-	85748	204835	374274	145849	203465	158921	229996	151464	230802
6.27_511.1183m/z	65	3	M+	C17H28N4O8P2S	0,44	74831	156869	458359	64968	115704	60261	82183	112126	184409
7.55_144.0477m/z	45	1	M+H	C6H9NOS	-4,10	58960	30949	166575	12389	61710	22174	95025	24352	106568
5.75_407.2292m/z	-	5	M+H	-	-	58296	189480	285766	136241	191154	80060	114160	159584	191208
4.42_377.5524m/z	64	3	M+2H	C22H30N9O13P3S	0,83	54863	101654	249337	86415	253599	151887	362374	109976	221374
7.84_327.1496m/z	-	5	M+H	-	-	54430	34594	150989	12087	100766	10396	85986	25118	102465
4.84_269.1245m/z	25	3	M+H	C11H16N4O4	-1,54	54295	70791	116967	21022	41479	32355	77947	45462	65986
4.68_251.1664m/z	-	5	M+2H	-	-	50262	58980	129137	108652	340397	24362	109837	186889	368983
5.72_385.7173m/z	-	5	M+2H	-	-	49714	143627	248427	186141	372517	53621	135101	235276	270238
3.04_224.0951m/z	-	5	M+H	-	-	49260	91870	177706	25867	49902	43308	82230	10789	21889
5.08_267.1452m/z	53	3	M+H	C12H18N4O3	-1,83	48659	22960	174538	17963	182167	14663	138715	18956	208199
8.63_373.7135m/z	-	5	M+2H	-	-	45581	1228	1624	112	788	257	406	88125	271229
6.55_431.1516m/z	67	3	M+	C17H27N4O5PS	-0,53	42319	106466	337084	19050	73298	15852	45241	72724	210768
9.08_491.7518m/z	-	5	M+2H	-	-	42280	129153	210679	229964	365764	84621	284282	174450	155577
8.83_405.7347m/z	-	5	M+2H	-	-	38680	44903	64697	39009	88628	17314	28215	106997	187917
5.61_279.6208m/z	-	5	M+2H	-	-	38631	7462	17200	19115	65860	8975	34203	20139	52026
1.94_291.6536m/z	-	5	M+2H	-	-	37907	70457	137898	177925	306795	39309	137759	108782	159350
8.76_564.3230m/z	-	5	M+2H	-	-	37526	25200	56774	26584	176160	3533	43100	38044	109738
1.79_209.1032m/z	52	3	M+H	C9H12N4O2	-3,03	36341	44710	171076	30874	109246	24223	138396	50072	214781
6.23_263.0961m/z	41	1	M+H	C12H14N4OS	-2,12	35003	10845	137814	10483	78889	11186	107384	12156	100852
4.99_330.2013m/z	-	5	M+2H	-	-	34394	29079	76151	30302	140941	8048	46000	92272	250277
8.30_415.7116m/z	-	5	M+2H	-	-	34309	83989	140484	162037	300611	46151	151880	193224	232956
4.76_279.6773m/z	-	5	M+2H	-	-	32428	49259	84642	72696	226200	22943	76639	136585	257060
6.81_460.7207m/z	-	5	M+2H	-	-	31118	38589	100239	45733	197572	4488	37824	41754	122196
5.65_338.5182m/z	-	5	M+H	-	-	30716	87945	148795	81074	215266	26953	93285	78437	162463
8.98_479.7429m/z	-	5	M+2H	-	-	29977	2586	7497	3899	62380	1101	17870	50043	148297
5.29_432.2278m/z	-	5	M+H	-	-	29095	29743	80317	4472	43833	2117	12058	68454	210291
7.68_424.1806m/z	-	5	M+H	-	-	27931	198460	122021	26041	2782	24793	3051	43480	7987
9.08_559.3209m/z	-	5	M+2H	-	-	27778	52826	105817	104059	255190	29761	140737	53469	91463
8.64_435.2121m/z	-	5	M+2H	-	-	25677	64846	108397	107093	265394	32087	139256	108017	151577
7.67_395.2171m/z	-	5	M+2H	-	-	25674	11076	74791	34399	134517	1104	13143	66458	142534
5.19_497.1030m/z	66	3	M+	C16H26N4O8P2S	1,01	24984	29693	124462	5206	14413	4105	6205	9899	26557
8.81_480.2335m/z	-	5	M+2H	-	-	23856	54708	113513	69204	194945	11869	81185	73208	122844
9.08_605.9692m/z	-	5	M+H	-	-	23777	57277	98680	11906	48318	8337	23639	68572	152624
5.26_373.2560m/z	-	5	M+H	-	-	23767	17865	46694	27402	133777	2052	29838	60247	152517
7.66_526.6204m/z	-	5	M+H	-	-	23429	21399	102053	39394	134522	920	12565	65853	134572
8.23_345.1918m/z	-	5	M+2H	-	-	23272	30604	68036	99621	207968	12026	82294	77017	94632
5.34_328.6776m/z	-	5	M+2H	-	-	23157	47566	111438	42015	150459	6427	55391	65863	133613
5.54_342.8812m/z	-	5	M+H	-	-	22503	17982	65850	17628	88824	1085	11166	47671	141093
9.08_559.5721m/z	-	5	M+2H	-	-	22134	43943	79496	86220	206733	21991	113614	37692	63912
6.44_343.1978m/z	-	5	M+H	-	-	22094	20570	63563	14180	63395	1350	10059	63804	140952
1.66_279.1275m/z	-	5	M+H	-	-	20633	1360	3434	1216	26322	486	13345	809	19527
5.43_410.1594m/z	-	5	M+H	-	-	20556	5411	16804	3788	13481	4197	15878	11032	23952
2.38_317.2188m/z	-	5	M+H	-	-	20191	811	418	0	0	0	0	11353	74727
8.48_349.1695m/z	-	5	M+H	-	-	19118	34440	89954	6086	44503	7732	41564	20751	86793
9.13_450.7082m/z	-	5	M+2H	-	-	18998	32934	61172	3786	40140	2190	13963	51026	157677
7.77_133.0682m/z	-	5	M+H	-	-	18565	40211	72567	19501	32892	24960	44558	16673	29489
8.55_425.8878m/z	-	5	M+2H	-	-	18313	8469	21834	81476	128975	25547	64972	68425	86919
8.67_538.7325m/z	-	5	M+2H	-	-	17071	39115	64508	3423	26643	5387	11944	40810	118401
6.94_382.2147m/z	-	5	M+2H	-	-	16455	229	1918	16266	93405	596	21564	25592	43725
7.67_395.4679m/z	-	5	M+2H	-	-	16125	4072	43302	15081	81504	0	6276	41169	86098
8.81_556.9466m/z	-	5	M+H	-	-	15638	49066	74049	34411	71594	19538	43914	66399	100860
8.14_429.7511m/z	-	5	M+2H	-	-	15269	9400	38028	13236	51942	637	4049	54075	94372
5.44_417.1359m/z	68	3	M+	C16H25N4O5PS	0,78	13861	13277	92369	2738	20245	968	9938	21986	73645
6.81_471.7117m/z	-	5	M+2H	-	-	13639	5090	21891	12573	83546	382	7700	12304	46735
5.54_353.1952m/z	-	5	M+2H	-	-	13405	22473	53441	4250	23486	1982	3660	20598	55925
4.52_181.1083m/z	49	1	M+H	C8H12N4O	-3,48	13169	19792	53999	20048	63509	31400	81259	22857	67611
4.76_433.7392m/z	-	5	M+2H	-	-	12657	18239	55224	15538	79363	1980	23790	28563	67983
6.25_237.2075m/z	-	5	M+H	-	-	12600	7356	12908	35560	40075	12408	25232	18315	19380
4.10_265.1117m/z	-	5	M+H	-	-	12539	460	41111	204	13403	90	9962	337	19579
7.68_526.9547m/z	-	5	M+H	-	-	12414	3573	46225	17660	65230	184	4932	34255	53948
4.38_301.1515m/z	-	5	M+H	-	-	12182	18677	75613	82	137	336	1635	117	934
5.55_343.2153m/z	-	5	M+H	-	-	12078	8998	35068	9698	49177	322	5780	24012	72185
8.94_605.9714m/z	-	5	M+H	-	-	11764	26100	45437	21160	69923	2922	18123	39139	62325
2.08_202.0530m/z	-	5	M+2H	-	-	11514	3161	10099	2552	12241	20171	74986	3459	12454
8.67_646.8552m/z	-	5	M+2H	-	-	10912	14697	40402	15320	46516	1929	9410	27499	66415
8.82_524.7411m/z	-	5	M+2H	-	-	10610	3062	5621	2020	12610	893	2827	13695	52108
4.51_265.1114m/z	-	5	M+H	-	-	10397	1084	41680	694	18548	822	13068	1728	37060
9.01_674.3871m/z	-	5	M+H	-	-	10330	22261	51191	10159	61484	1626	23498	29989	77419
